# Absence of Alpha-tACS Aftereffects in Darkness Reveals Importance of Taking Derivations of Stimulation Frequency and Individual Alpha Variability Into Account

**DOI:** 10.3389/fpsyg.2018.00984

**Published:** 2018-06-20

**Authors:** Heiko I. Stecher, Christoph S. Herrmann

**Affiliations:** ^1^Experimental Psychology Lab, Department of Psychology, European Medical School, Cluster for Excellence “Hearing for all”, Carl von Ossietzky University, Oldenburg, Germany; ^2^Research Center Neurosensory Science, Carl von Ossietzky University, Oldenburg, Germany

**Keywords:** transcranial alternating current stimulation (tACS), EEG, aftereffect, alpha oscillations, replication, tES reliability

## Abstract

Transcranial alternating current stimulation (tACS) has found widespread use as a basic tool in the exploration of the role of brain oscillations. Many studies have shown that frequency-specific tACS is able to not only alter cognitive processes during stimulation, but also cause specific physiological aftereffects visible in the electroencephalogram (EEG). The relationship between the emergence of these aftereffects and the necessary duration of stimulation is inconclusive. Our goal in this study was to narrow down the crucial length of tACS-blocks, by which aftereffects can be elicited. We stimulated participants with α-tACS in four blocks of 1-, 3-, 5-, and 10-min length, once in increasing and once in decreasing order. After each block, we measured the resting EEG for 10 min during a visual vigilance task. We could not find lasting enhancement of α-power following any stimulation block, when comparing the stimulated groups to the sham group. These findings offer no information regarding the crucial stimulation duration. In addition, this conflicts with previous findings, showing a power increase following 10 min of tACS in the alpha range. We performed additional explorative analyses, based on known confounds of (1) mismatches between stimulation frequency and individual alpha frequency and (2) abnormalities in baseline α-activity. The results of an ANCOVA suggested that both factor explain variance, but could not resolve how exactly both factors interfere with the stimulation effect. Employing a linear mixed model, we found a significant effect of stimulation following 10 min of α-tACS in the increasing sequence and a significant effect of the mismatch between stimulated frequency and individual alpha frequency. The implications of these findings for future research are discussed.

## Introduction

Transcranial alternating current stimulation, in which weak electrical oscillating currents are administered by electrodes placed directly on the scalp, has emerged as a non-invasive technique for brain stimulation. Its role as a tool in clinical therapy and basic brain research is being investigated, as it is believed to interact directly with endogenous brain oscillations (Antal and Paulus, 2013; Herrmann et al., 2013). This could enable the direct exploration of their functional role (Thut et al., 2012). tACS has been shown to successfully alter behavioral processes like cognition (Vosskuhl et al., 2015; Kasten and Herrmann, 2017), perception (Feurra et al., 2011; Helfrich et al., 2014a; Strüber et al., 2014), motor behavior (Feurra et al., 2013) and ongoing oscillations (Helfrich et al., 2014b; Neuling et al., 2015; Ruhnau et al., 2016). It has been postulated that tACS directly interferes with endogenous oscillations by entrainment (Thut et al., 2011; Reato et al., 2013; Herrmann et al., 2016).

Aside from online-effects (occurring ‘during’ the stimulation) many studies have also shown physiological aftereffects, persisting even after the stimulation has ended (see Veniero et al., 2015). The exact nature of these aftereffects is unclear, and Vossen et al. (2015) it has been shown that these aftereffects are not likely to be a manifestation of entrainment. It has been suggested, that they are caused by spike time dependent plasticity (STDP) (Zaehle et al., 2010; Vossen et al., 2015), causing long-term-potentiation (LTP) or long-term-depression (LTD). The α-band in the electroencephalogram (EEG) is a frequency in which robust aftereffects of power-enhancement have been found. Effects have been found following 10-min (Zaehle et al., 2010) and 20-min (Neuling et al., 2013) of tACS at individual alpha frequency (IAF). It has been shown that these aftereffects persist for up to 70 min post-stimulation (Kasten et al., 2016). Comparable effects were also observed with intermittent protocols of a cumulative length of 11–15 min, if the single trains had a duration of at least 8 s (Vossen et al., 2015). By contrast, intermittent protocols of 1-s trains and a cumulative duration of 10 min did not yield any effects (Strüber et al., 2015). As of yet, the duration (and amplitude) of α-tACS required to produce lasting physiological effects is unknown. However, dependency on duration is implied if the aftereffect originates from synaptic strengthening, due to LTP/LTD, between the relevant neuronal networks. An understanding of the duration and the occurrence of lasting effects is essential for future experimental protocols and for dosages for therapeutic approaches.

In this study, we intended to find the range of crucial α-tACS durations necessary for the elicitation of measurable aftereffects, by observing the band-power in the EEG following tACS-blocks of different lengths, in a sham-controlled study. To this end, we employed an exploratory cascade design of increasing durations of α-stimulations. In order to partially control for effects of time and carry-over effects of one block to the next, we also used a reverse sequence. Since 10 min of tACS has been shown to elicit aftereffects in the α-band (Zaehle et al., 2010), we used a 10-min block of stimulation as a starting point. This enabled the study to serve as a replication of the results found by Zaehle et al. (2010). Sleep studies utilizing 5-min of δ-oscillatory direct current stimulation (otDCS) were also successful in eliciting short-lasting aftereffects (Marshall et al., 2006; Garside et al., 2014). These results suggest that 5-min might be a promising duration where aftereffects in the α-band are still measurable. Additionally, we tested 3- and 1-min durations. To look for immediate short-lasting effects, we included a 10-min observation window of EEG following each application of stimulation. We hypothesized that we would find at least one observation window, where the power is significantly more enhanced than in the sham condition.

## Materials and Methods

### Participants

Fifty right-handed volunteers, who reported no neurological or psychiatric disorders, aged 18–30 (25 ♀) participated in the study. All participants had normal or corrected-to-normal vision and were recruited from the student body of the Carl von Ossietzky University Oldenburg. All gave written consent and received a monetary compensation for their participation. The design of the study was approved by the ethics committee (“Komission für Forschungsfolgenabschätzung und Ethik”) of the Carl von Ossietzky Universität Oldenburg and was in accordance with the declaration of Helsinki. Due to technical problems, the data of five participants was discarded from the analysis and the measurements were redone with new participants. To each stimulation group, 15 participants were assigned, while 15 participants received sham-stimulation. During the analysis, one additional participant showed an average increase in α-power exceeding 4 σ of the total sample’s *z*-scored values and was excluded from the statistical analysis. The resulting sham group (*N* = 14, 8 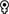) had an average age of 23.8 years (±3.6). The stimulation group with an increasing sequence (*N* = 15, 8 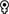) had an average age of 24.0 years (±2.4), while the stimulation group with a decreasing sequence (*N* = 15, 8 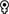) had an average age of 23.8 years (±2.8).

### EEG

**FIGURE 1:**
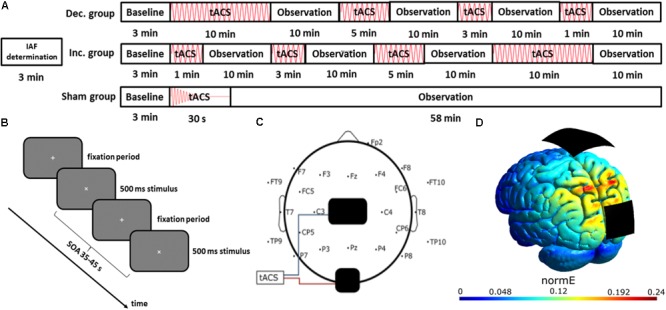
Experimental *s*etup. **(A)** Time course of the experiment: the IAF of each participant was determined in a 3-min resting EEG. Afterwards, participants of all groups had to conduct a visual vigilance task for 58 min, while they received either sham stimulation or four blocks of stimulation in decreasing or increasing sequence, each followed by a 10-min window of no-stimulation. **(B)** Visual Vigilance task: Each participant had to fixate a small white cross in the center of a gray screen. Every 35–45 s, the fixation cross was rotated by 45° for 500 ms, and the participants had to respond by pressing a button using their right index finger. **(C)** Electrode configuration: EEG was recorded using 23 electrodes, placed according to the international 10–10 system, referenced against Fp1. tACS electrodes were placed at Cz and Oz. **(D)** Current simulation using SIMNIBS: simulation of the stimulation’s electric field strength, covering the posterior brain areas.

The EEG data was acquired at an acquisition rate of 10 kHz, using an actiCHamp amplifier (Brain Products GmbH, Gilching, Germany) with 23 active electrodes. The electrodes were placed according to the international 10–10 system, omitting the sites of the stimulation electrodes (see **Figure 1C**). Fp1 served as reference. A vertical EOG-channel was recorded by one electrode placed under the right eye. Pycorder software (Brain Products GmbH, Gilching, Germany) was used for recording. All impedances were below 10 kΩ before starting the experiments.

### Electrical Stimulation

Transcranial alternating current stimulation was administered in accordance with previous studies (Neuling et al., 2013; Kasten et al., 2016; Stecher et al., 2017), with a maximum posterior stimulation [simulated using SimNIBS 2.0 (Thielscher et al., 2015); see **Figures 1C,D**]. This constitutes a different montage to 10-min α-tACS study of Zaehle and colleagues, who used a PO9/PO10-montage. We employed a Neuroconn DC Plus Stimulator (Neuroconn, Ilmenau, Germany) and two carbonized rubber electrodes, sized 5 cm × 7 cm and 4.5 cm × 4.5 cm. The smaller electrode was placed at Oz and the larger one at Cz. They were fixed to the scalp using Ten20 conductive paste (D.O. Weaver, Aurora, CO, United States). It was ensured that impedances were below 10 kΩ, before participants received a stimulation current at 1 mA to confirm they experienced neither pain nor irritation. From experience, this intensity is also below the threshold for phosphenes in the employed electrode configuration, although participants were not specifically asked about them and none gave any indication of perceiving any phosphenes. The sinusoidal stimulation signal was computed in MATLAB 2012b (The MathWorks Inc., Natick, MA, United States) and generated by a digital-to-analog converter (DAQ NI USB 6229, National Instruments, Austin, TX, United States), which drove the stimulator via remote access. The total duration of stimulation was 18 min. In the sham condition, the stimulation was faded out to 0 mA after 30 s. The stimulation protocol differed from previous studies (Zaehle et al., 2010; Neuling et al., 2013; Kasten et al., 2016) by employing a fixed amplitude of 1 mA in contrast to using a sub-sensation-threshold stimulation.

### Experimental Procedure

At the start of each session, participants were informed and the tACS and EEG electrodes were prepared. After preparation, participants were told to keep their eyes open and to relax, while a 3 min resting EEG was recorded. From this recording the individual alpha frequency was determined by computing the peak frequency between 7.5 and 12 Hz in the raw recording of electrode Pz. For this determination no filtering or artifact-processing was applied.

During the main experiment, the participants were seated in a dark room, with a monitor as a sole light source. To maintain a stable level of vigilance, participants had to conduct a visual vigilance task, which required them to fixate a white cross on a monitor, and respond to a 500 ms rotation of the cross by pressing a button with their right index finger (**Figure 1B**). This visual vigilance task was in accordance with previous studies on α-tACS aftereffects (Zaehle et al., 2010; Vossen et al., 2015; Kasten et al., 2016; Stecher et al., 2017). The main experiment consisted of a 3 min baseline and four stimulation blocks of varying length, each followed by a 10 min observation block (see **Figure 1A**). The stimulation block sequence was 1-, 3-, 5-, and 10-min in the increasing-sequence-group and in the reverse order for the decreasing-sequence group.

### Data Analysis

Data processing was carried out using MATLAB 2012b and the Fieldtrip toolbox (Oostenveld et al., 2011). The continuous EEG data was down-sampled to 1000 Hz, high-pass filtered above 0.5 Hz and low-pass filtered below 48 Hz. EEG data was then cut into segments starting 30 s after stimulation and ending 30 s before stimulation, resulting in a 3 min baseline block and four segments of 9 min length for both stimulation groups. For both stimulation groups, corresponding parts of the data from the sham group were selected. The data was then re-referenced to combined Fp1/Fp2 electrodes to prevent lateralization of effects due to the asymmetrical reference site during the recording and then further subdivided into 1-s trials. These trials were then used in an ICA approach for the manual removal of components containing vertical or horizontal eye-movements. Trials containing voltage differences of more than 200 μV were rejected as artifacts to clear out DC- distortions and strong muscle-artifacts. The first 66% of artifact free trials of each segment were used to compute the mean α-power (IAF ± 2 Hz as determined in the last post-stimulation segment) for each block using a Hanning window with 2-s zero padding. This percentage was the minimal number of trials, necessary to avoid omitting further participants. For post-stimulation power analysis, the data of the four post-stimulation segments were then normalized to the power in the baseline-segment.

### Statistics

Statistical analysis was performed by using MATLAB, SPSS 24.0 (IBMCorp, Armonk, NY, United States) and the software package R 3.3.0 (R Foundation for Statistical Computing, Vienna, Austria) employing the nmle-package (Pinheiro et al., 2016) and the piecewise SEM-package (Lefcheck, 2016). The combined stimulation groups were tested against the sham group for differences in adverse effects by using a Wilcoxon–Mann–Whitney-*U* test. Awareness of stimulation was tested by using a Chi-squared test. For accuracy and reaction times in the vigilance task, the stimulation groups were pooled and tested against the sham group with a two-sided *t*-test. Accuracy and reaction times were evaluated using ANOVAS with the 3- level factor group. Groups were checked for differences in baseline α-power by employing Mann–Whitney *U* tests. The change of α-power post-stimulation was tested by employing a repeated measures ANOVA with the between subject factor group (stim/sham) and the within-factor time (observation windows 1, 2, 3, 4) for both stimulation groups against the corresponding time-segments of the sham group. All *p*-values were Greenhouse-Geisser corrected, when the assumption of sphericity was violated.

## Results

### Behavioral Results

Stimulation did not cause side effects or behavioral differences in the vigilance task: rating of the adverse effects of tACS did not differ between the pooled stimulation groups and the sham group (all *p* > 0.05). Participants of the stimulated groups did not think they were stimulated more frequently than sham-participants (stim: 12.12%, sham: 23.53%, χ^2^_1_ = 1.086, *p* = 0.297). Neither accuracy nor reaction times in the vigilance task showed differences between stimulation and the sham group (accuracy: *t*_42_ = 0.248, *p* = 0.805; reaction times: *t*_42_ = -0.506, *p* = 0.615).

### EEG Results

#### Standard Analysis

**FIGURE 2:**
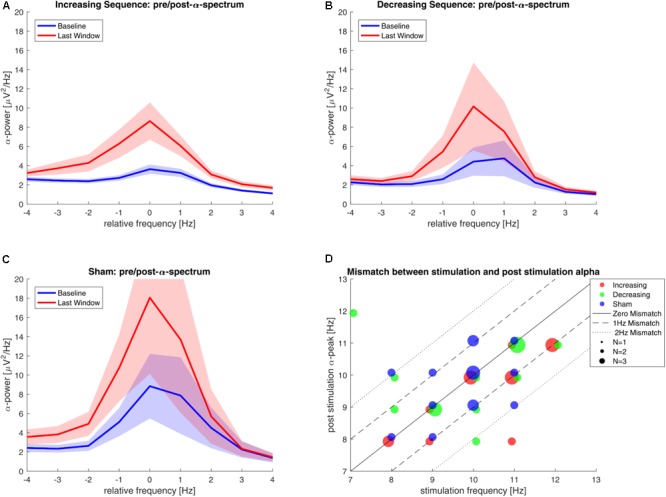
Parietal power-spectra in the α-range before stimulation and at the end of the recording and mismatch between stimulated frequency and individual alpha frequency. **(A–C)** Mean posterior alpha power for the increasing-sequence group, decreasing-sequence group, and sham-group. Power is taken from the baseline period (blue) and from the last 9 min of the recording (red). The frequency axis is centered around IAF as determined in the last 9 min window. Shaded areas show the standard error of the mean. **(D)** Frequency mismatch scatterplot: The stimulation frequency vs. the ‘true’ IAF as determined in the last 9 min of recording is plotted. The dot size denotes number of participants. The solid line marks the zero-mismatch diagonal. Dashed and dotted lines mark the areas of ±1 Hz and ±2 Hz.

The baseline α-power neither differed between the increasing-sequence (median = 2.651) and the sham group (median = 2.704), as tested with a Wilcoxon–Mann–Whitney-*U* test [Z = -0.414, *p*(uncorrected) = 0.678], nor between the decreasing-sequence (median = 1.745) and the sham group (median = 2.704) [*Z* = -0.720, *p*(uncorrected) = 0.472]. The baseline power is plotted in **Figures 2A–C** for all groups (blue lines), relative to the IAF as determined from the last post-stimulation EEG-segment (see below). The individual spectra for all participants can be found in the Supplemantary Figures S2–S4.

As the IAF can show variability within participants and the initial determination can be faulty (Vossen et al., 2015; Stecher et al., 2017), we checked if the individual stimulation frequency (ISF) as determined before the stimulation matched the IAF after stimulation. We calculated the mismatch between the ISF and the alpha peak in the last observational window, which we consider the ‘true’ IAF for every participant (see **Figure 2D**). The ISF and IAF only matched in 20 out of 44 participants.

**FIGURE 3:**
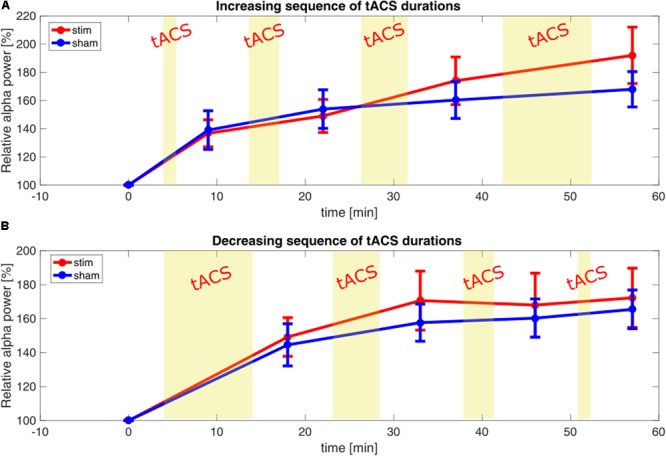
Relative parietal α-power post-stimulation. **(A)** Time-course of α-power relative to baseline, comparing increasing-sequence stimulation group (red) and sham (blue). Each point represents the average power of a 9-min observation window. Yellow bars represent blocks of stimulation. Error bars depict the standard error of the mean. **(B)** Time-course of α-power relative to baseline, comparing decreasing-sequence stimulation group (red) and sham (blue): each point represents the average power of a 9-min observation window. Yellow bars represent blocks of stimulation. Error bars depict standard error of the mean.

Post-stimulation effects were analyzed using a standard approach like in comparable studies (Neuling et al., 2013; Kasten et al., 2016). A Shapiro–Wilk test showed that neither the data of the increasing-sequence (0.876, *p* < 0.001) nor the decreasing-sequence (0.949, *p* < 0.001) was normally distributed. We employed an ANOVA in absence of a non-parametric equivalent, even though sample size of *n* < 30 is normally not assumed to be robust against such a violation. We used two repeated measures ANOVAs to test the increasing-sequence tACS group and the decreasing-sequence tACS groups independently against the sham group. In the comparison of the increasing-sequence and the sham groups, we found a main effect of time (*F*_3,81_ = 14.031, *p* < 0.001, η^2^ = 0.342), whereas the factor group (*F*_1, 27_ = 0.174, *p* = 0.680, η^2^ = 0.006) and the interaction time × group (*F*_3,81_ = 1.950, *p* = 0.151, η^2^ = 0.067) remained non-significant. In the comparison of the decreasing-sequence and the sham groups, we also found a significant main effect of time (*F*_3,81_ = 7.010, *p* = 0.002, η^2^ = 0.206), and no significant effects of the factor group (*F*_1,27_ = 0.1728, *p* = 0.682, η^2^ = 0.006) and the interaction time × group (*F*_3,81_ = 0.233, *p* = 0.794, η^2^ = 0.009). The general increase in relative α-power for both the tACS and the sham groups can be seen in **Figure 3** (confer with Supplementary Figure S1 in the Supplementary Material, showing no short-term effects for smaller time-windows). The relative power of each EEG-windows of both tACS groups is plotted with the power of the respective windows of the sham-group. Note that the increase seems to be limited to the alpha-band range (see **Figures 2A–C**).

#### Exploratory Analysis

Due to unexplained discrepancies between published reports and the results of our standard analysis approach, we performed an additional analysis to uncover confounding factors. Previous tACS studies in the α-range show that the power-enhancement relative to sham correlated with the negative mismatch between the stimulated frequency and true IAF (Vossen et al., 2015). Additionally it could be shown, that the inclusion of such a mismatch as a factor explains observed variance when modeling power-enhancement (Stecher et al., 2017). The large variance in the baseline α-power (see **Figures 2A–C**, albeit not significantly different between groups), encouraged us to test, whether baseline-power might influence the capacity for post-stimulation enhancement. For this reason, we included both the factors *frequency mismatch* as well as *baseline power* as covariates to a repeated measure ANCOVA. This did not lead to different results in the case of the decreasing sequence condition compared to sham, revealing no significant main effect of time [*time* (*F*_1,75_ = 1.767, *p* = 0.180, η^2^ = 0.066)], no significant effects of the factor *group* (*F*_1,25_ = 0.199, *p* = 0.659, η^2^ = 0.008), or the interaction *time × group* (*F*_3,75_ = 0.578, *p* = 0.570, η^2^ = 0.023). In the case of the increasing sequence, however, the inclusion of the covariates not only revealed the above-mentioned significant main effect of *time* (*F*_1,75_ = 6.471, *p* = 0.018, η^2^ = 0.206), but also a significant interaction of *time × group* (*F*_3,75_ = 4.134, *p* = 0.009, η^2^ = 0.142). The interaction of *time* × *basepower* showed a trend (*F*_3,75_ = 2.703, *p* = 0.051, η^2^ = 0.098), while the factor group (*F*_1, 25_ = 0.931, *p* = 0.344, η^2^ = 0.036) and the interaction *time* × *mismatch* did not reach significance (*F*_3,75_ = 1.478, *p* = 0.227, η^2^ = 0.056).

However, the resolution of the interaction *time* × *group*, employing *post-hoc* one-way ANCOVAs for every timepoint between groups, did not yield any significant differences between groups at any timepoint (T1 *group*: *F*_1,25_ = 0.031, *p* = 0.862, η^2^ = 0.001; T2 *group*: *F*_1,25_ = 0.148, *p* = 0.704, η^2^ = 0.006; T3 *group*: *F*_1,25_ = 0.1966, *p* = 0.173, η^2^ = 0.073; T4 *group*: *F*_1,25_ = 2.452, *p* = 0.130, η^2^ = 0.89; all *p*-values uncorrected).

We then tested if a random mixed effect model, which allows inter-subject variability would be better suited to explain our results. Initially we created a saturated model that predicted alpha power from the fixed effects of 9 *time points* per post-stimulation window, 4 *blocks*, 2 *groups* and effects of *frequency-mismatch, basepower* as well as their interactions and random effects for each participants ID. This did not yield any significant factors and the high-level interactions would be hard to interpret. Therefore, we omitted the factor of time and started with a minimal model, which only contained the hypothesis-relevant factors *block* (post-stimulation window) and *group* (tACS or sham). Thereby the model is equivalent to the initial ANOVA, but allowed a random effect of participant’s *ID*. To this minimal model, we added effects of the factors *mismatch* and *basepower* as different combinations with the other two factors and compared the Akaike Information Criterion of the resulting model to the minimal model. For the increasing sequence comparison, a model containing an interaction of *block* and *mismatch*, described by equation (1) resulted in a lower AIC that the minimal model.

(1) $$\begin{array}{lll} \alpha & = & \beta_{0}+\beta_{1}\;\text{group}1\;+\;\beta_{2}\;\text{block}2\;+\;\beta_{3}\;\text{block}3\;+\;\beta_{4}\;\text{block}4\\ & & +\;\beta_{5}\;\text{group}1*\text{block}2\;+\;\beta_{6}\;\text{group}1*\text{block}3\\ & & +\;\beta_{7}\;\text{group}1*\text{block}4\;+\;\beta_{8}\;\text{block}\;:\;\text{mismatch}\;+\;\gamma_{0,\text{ID}}\;+\;\varepsilon \end{array}$$

For the decreasing-sequence comparison, all additions to the minimal model resulted in an increase in AIC, so that the minimal model (2) was chosen for further analysis.

(2) $$\begin{array}{lll} \alpha & = & \beta_{0}+\beta_{1}\;\text{group}1\;+\;\beta_{2}\;\text{block}2\;+\;\beta_{3}\;\text{block}3\;+\;\beta_{4}\;\text{block}4\\ & & +\;\beta_{5}\;\text{group}1*\text{block}2\;+\;\beta_{6}\;\text{group}1*\text{block}3+\;\beta_{7}\;\text{group}1*\text{block}4\\ & & +\;\gamma_{0,\text{ID}}\;+\;\varepsilon \end{array}$$

The resulting equations (1) and (2) predict the α-power for the fixed effects β, the random effects γ and the residual error 𝜀. The estimators of the final model for the increasing-sequence condition are listed in **Table 1**, showing a significant effect of the factor *block* at the levels 2, 3, and 4, denoting a general increase in alpha power over time. The significant interactions of the stimulation group with the fourth block, implies a significant increase in α-power following 10 min of α-tACS. The significant interaction of *mismatch* and *block* represents a negative slope of α-power increase over blocks, due to large mismatches. In **Table 2**, the results of the decreasing-sequence condition are shown. While a significant effect of the factor *block* on α-power can be seen, the factor *group* has no effect.

**Table 1:** Increasing transcranial alternating current stimulation (tACS)-sequence: result summary of linear mixed effect model.

Parameter	Coefficients β	*SE*(β)	*t*	*p*
(β_0_) Intercept	145.019	17.709	8.189	<0.001
(β_1_) Group1	0.021	25.092	0.001	0.999
(β_2_) Block2	17.489	6.042	8.894	0.004
(β_3_) Block3	32.302	6.042	5.346	<0.001
(β_4_) Block4	45.992	6.042	7.611	<0.001
(β_5_) Group1:Block2	2.899	8.686	0.334	0.739
(β_6_) Group1:Block3	13.656	9.092	1.502	0.134
(β_7_) Group1:Block4	25.443	9.733	2.614	0.009
(β_8_) Mismatch:Block	–4.618	2.243	–2.056	0.040

*Coefficient estimates β for the fixed effects, standard Error SE(β), t-value t and significance level p. The model’s has marginal R^*2*^ of 0.074 and a conditional R^*2*^ of 0.669.*

**Table 2:** Decreasing tACS-sequence: result summary of linear mixed effect model.

Parameter	Coefficients β	*SE*(β)	*t*	*p*
(β_0_) Intercept	157.086	17.118	9.177	<0.001
(β_1_) Group1	–7.063	5.096	–0.302	0.765
(β_2_) Block2	15.761	5.096	3.093	0.020
(β_3_) Block3	25.778	5.096	5.058	<0.001
(β_4_) Block4	29.945	5.096	5.876	<0.001
(β_5_) Group1:Block2	–0.512	6.963	–0.074	0.941
(β_6_) Group1:Block3	–4.837	6.963	–0.695	0.487
(β_7_) Group1:Block4	–5.525	6.963	–7.794	0.428

*Coefficient estimates β for the fixed effects, standard Error SE(β), t-value t and significance level p. The model’s has marginal R^*2*^ of 0.025 and a conditional R^*2*^ of 0.713.*

## Discussion

### General Discussions and Discrepancies

When we used the standard statistical approach, our results showed no significant effect of stimulation on post-stimulation power in the alpha band, neither in an increasing nor in a decreasing sequence of stimulation durations. Only a general increase of power over time was found, as was expected for a long monotonous task in darkness. We would have expected to replicate previous findings of a power increase following 10 min of α-tACS (Zaehle et al., 2010) with a subsample of our data. The first post-stimulation measure in the decreasing-sequence conditions strongly mimics the setup of Zaehle et al. (2010), despite the different montage of stimulation electrodes. Taking the effect sizes from their results into considerations (one-sided *t*-test on post-stimulation α-power, with a desired statistical power of 80% results in a sample size of 15 participants per group) our sample size should have been sufficiently large to expect a significant effect of stimulation at the first time-point for the respective group. One possible explanation for the discrepancy might be that the effect sizes in previously published α-tACS studies with small samples were overestimated, which would leave our study severely underpowered.

Another possible explanation is that our protocol was altered from the established procedures by unconsidered factors. When compared to other studies in our lab that employed a similar task (Zaehle et al., 2010; Neuling et al., 2013; Kasten et al., 2016), the natural increase of α-power within our unstimulated group is remarkably high. Indeed neither Neuling et al. (2013) nor Zaehle et al. (2010) found a significant increase in the α-power within the sham groups, while Kasten et al. (2016) found a mean increase by 40% only after 90 min post-stimulation – a value, which was already reached as early as 8 min post-stimulation in our experiment. When looking for systematic differences in the setups, we noticed that our experiment was conducted in complete darkness with the monitor as the sole source of light in the laboratory, whereas the setups of the aforementioned studies (Zaehle et al., 2010; Neuling et al., 2013; Kasten et al., 2016) included ambient light sources. In a recent study (Stecher et al., 2017), we could show that the level of ambient light significantly influences the rise of alpha power within the first 25 min of recording, while stimulation-related effects only emerged after that. Thus, we suggest that in the case of the current study any tACS-induced aftereffects in the early stimulation blocks might have been masked by the darkness-induced huge increase in α-power. In the decreasing-sequence condition, this likely could have prevented the replication of an aftereffect following the 10-min stimulation.

Additionally, our exploratory analysis employing covariates and a linear mixed effect model showed that ISF/IAF-mismatch and random differences in baseline α-power explain variance. The LMEM even showed a significant increase of α-power following 10 min of tACS, when employed as the last stimulation block. This finding may indicate that both mismatch and individual variability in alpha power pose potential confounds, individually influencing the post-stimulation development of α-power. Our results suggest that the standard approach of repeated measures ANOVAs and ANCOVAs may not always be the best choice for small datasets, and that data showing high inter-individual variability might be explored better by using mixed effect models.

It is unclear, which tACS duration at IAF and 1mA is necessary to elicit aftereffects, but our results indicate that future studies need to be designed in a way that better controls for confounding factors.

### Limitations and Points to Consider

Other researchers have already discovered how individual brain anatomy might influence the efficiency of non-invasive brain stimulation (Krause and Cohen Kadosh, 2014; Veniero et al., 2017). Therefore, tACS studies should, whenever possible, consider the individual anatomy, taken from neuroimaging approaches, for precise placement of electrodes and choice of stimulation parameters (Bergmann et al., 2016). We think three limitations inherent in our design illustrate the importance of additional points to consider in future research:

First limitation: Minor differences in our environmental factors might have had a large independent effect on our measured outcome variable. We did not consider differences in environmental illumination when designing our study as a partial replication of previous results (Zaehle et al., 2010). Especially when studying α-activity, it is important to recognize all additional factors, which might independently induce changes, such as illumination (Min et al., 2013; Stecher et al., 2017), task induced fatigue (Cajochen et al., 1995; Oken et al., 2006) or memory load (Jensen, 2002; Tuladhar et al., 2007). All protocols should incorporate stable, replicable conditions with minimal influence on the measured outcome-variable and the states of the stimulated networks should be carefully considered (Fertonani and Miniussi, 2016). Additionally, as the control treatment consisted of a sham-stimulation it is unclear if the perception of consistent stimulation might have altered the behavior in the stimulated groups. This could be circumvented in future studies by employing control frequencies, which can also prove the frequency-specificity of tACS.

Second limitation: Since it is believed that the aftereffects of tACS are caused by LTP/LTD-processes due to entrainment during stimulation (Zaehle et al., 2010; Vossen et al., 2015), the physics of entrainment (Pikovsky et al., 2003) require a close frequency-match between endogenous oscillation and the exterior driving frequency. Even though our protocol involved an adjustment of the stimulation frequency to the IAF, our *post hoc* analysis revealed that we missed the right frequency in nearly half the cases, with maximum deviations of up to 5 Hz (mean deviation in stimulated groups: 0.7 Hz). This poor estimation is probably caused by the short and unprocessed resting recording that we employed to find the posterior α-peak. Future studies should take better care in finding the true IAF by using a longer recording, and employing advanced methods for the processing of EEG-data, like basic artifact rejection and independent component analysis. The overall information regarding the relationship between successful stimulation and matching the IAF is quite sparse; only two studies so far have looked into eventual mismatches (Vossen et al., 2015; Stecher et al., 2017). Therefore, it might prove beneficial for future tACS studies to execute *post hoc* explorations of stimulation-frequency mismatches to get a better understanding of its effects.

Third limitation: The standard ANOVA as employed by previous studies (Neuling et al., 2013; Helfrich et al., 2014a; Vossen et al., 2015; Kasten et al., 2016) assumes small inter-individual variability in the distribution of α-power and enhancement. Additionally, confounding factors that might influence the susceptibility toward tACS are seldom explored, negating their capability to explain additional variance. Even though an ANOVA is often thought to be robust against violations of its general assumptions, it might be beneficial for some studies to use mixed-effect models that enable the modeling of the effects of additional factors while simultaneously allowing for more inter-individual variability.

## Author Contributions

HS and CH designed the study and wrote the article. HS acquired and analyzed the data.

## Conflict of Interest Statement

CSH has filed a patent application on brain stimulation and received honoraria as editor from Elsevier Publishers, Amsterdam. The remaining author declares that the research was conducted in the absence of any commercial or financial relationships that could be construed as a potential conflict of interest.
