# Corrigendum: Absence of Alpha-tACS Aftereffects in Darkness Reveals Importance of Taking Derivations of Stimulation Frequency and Individual Alpha Variability Into Account

**DOI:** 10.3389/fpsyg.2018.01769

**Published:** 2018-09-19

**Authors:** Heiko I. Stecher, Christoph S. Herrmann

**Affiliations:** ^1^Experimental Psychology Lab, Department of Psychology, European Medical School, Cluster for Excellence “Hearing for all”, Carl von Ossietzky University, Oldenburg, Germany; ^2^Research Center Neurosensory Science, Carl von Ossietzky University, Oldenburg, Germany

**Keywords:** transcranial alternating current stimulation (tACS), EEG, aftereffect, alpha oscillations, replication, tES reliability

In the original article, there was a mistake in Table 2 as published. The last parameter in the table is wrongly designated as “(β_8_) Group1:Block4” instead of “(β_7_) Group1:Block4.”

The corrected Table 2 appears below.

**Table 2:** Decreasing tACS-sequence: result summary of linear mixed effect model.

**Parameter**	**Coef. β**	**SE(β)**	***t***	***p***
(β_0_) Intercept	157.086	17.118	9.177	< 0.001
(β_1_) Group1	−7.063	5.096	−0.302	0.765
(β_2_) Block2	15.761	5.096	3.093	0.020
(β_3_) Block3	25.778	5.096	5.058	< 0.001
(β_4_) Block4	29.945	5.096	5.876	< 0.001
(β_5_) Group1:Block2	−0.512	6.963	−0.074	0.941
(β_6_) Group1:Block3	−4.837	6.963	−0.695	0.487
(β_7_) Group1:Block4	−5.525	6.963	−7.794	0.428

*Coefficient estimates β for the fixed effects, standard Error SE(β), t-value t and significance level p. The model's has marginal R^2^ of 0.025 and a conditional R^2^ of 0.713*.

In the results section, two decimal points are missing: the *F*-value of time x group interaction: *F*_3, 75_ = 578, is actually *F*_3, 75_ = 1.578. The *p*-value for T1: *p* = 862 is actually *p* = 0.862. In the same section in equations 1&2: the coefficient β_4_ is wrongly used twice: “β_4_ group1*block2,” should be “β_5_ group1*block2” and all subsequent “βs” numbered accordingly. A correction has been made to EEG Results, Exploratory Analysis, Paragraph 1 and Paragraph 2 and equation 1&2:

Due to unexplained discrepancies between published reports and the results of our standard analysis approach, we performed an additional analysis to uncover confounding factors. Previous tACS studies in the α-range show that the power-enhancement relative to sham correlated with the negative mismatch between the stimulated frequency and true IAF (Vossen et al., 2015). Additionally it could be shown, that the inclusion of such a mismatch as a factor explains observed variance when modeling power-enhancement (Stecher et al., 2017). The large variance in the baseline α-power (see Figures 2A–C, albeit not significantly different between groups), encouraged us to test, whether baseline-power might influence the capacity for post-stimulation enhancement. For this reason, we included both the factors *frequency mismatch* as well as *baseline power* as covariates to a repeated measure ANCOVA. This did not lead to different results in the case of the decreasing sequence condition compared to sham, revealing no significant main effect of time [*time* (*F*_1, 75_ = 1.767, *p* = 0.180, η2 = 0.066)], no significant effects of the factor

*group* (*F*_1, 25_ = 0.199, *p* = 0.659, η2 = 0.008), or the interaction *time* × *group* (*F*_3, 75_ = 0.578, *p* = 0.570, η2 = 0.023). In the case of the increasing sequence, however, the inclusion of the covariates not only revealed the above-mentioned significant main effect of *time* (*F*_1, 75_ = 6.471, *p* = 0.018, η2 = 0.206), but also a significant interaction of *time* × *group* (*F*_3, 75_ = 4.134, *p* = 0.009, η2 = 0.142). The interaction of *time x basepower* showed a trend (*F*_3, 75_ = 2.703, *p* = 0.051, η2 = 0.098), while the factor group (*F*_1, 25_ = 0.931, *p* = 0.344, η2 = 0.036) and the interaction *time x mismatch* did not reach significance (*F*_3, 75_ = 1.478, *p* = 0.227, η2 = 0.056).

However, the resolution of the interaction *time x group*, employing *post-hoc* one-way ANCOVAs for every timepoint between groups, did not yield any significant differences between groups at any timepoint (T1 *group*: *F*_1, 25_ = 0.031, *p* = 0.862, η2 = 0.001; T2 *group*: *F*_1, 25_ = 0.148, *p* = 0.704, η2 = 0.006; T3 *group*: F_1, 25_ = 0.1966, p = 0.173, η2 = 0.073; T4 *group*: F_1, 25_ = 2.452, *p* = 0.130, η2 = 0.89; all *p*-values uncorrected).

(1) $$\begin{array}{l} \alpha =\beta_{0}+\beta_{1}\;group1+\beta_{2}\;block2+\beta_{3}\;block3+\beta_{4}\;block4\\ +\beta_{5}\;group1*block2+\beta_{6}\;group1*block3\\ +\beta_{7}\;group1*block4+\beta_{8}\;block:mismatch{+\gamma }_{0,ID}+\varepsilon \end{array}$$

(2) $$\begin{array}{l} \alpha =\beta_{0}+\beta_{1}\;group1+\beta_{2}\;block2+\beta_{3}\;block3+\beta_{4}\;block4\\ +\beta_{5}\;group1*block2+\beta_{6}\;group1*block3\\ +\beta_{7}\;group1*block4+\;\gamma_{0,ID}+\;\varepsilon \end{array}$$

The authors apologize for these errors and state that this does not change the scientific conclusions of the article in any way.

The original article has been updated.

## Conflict of interest statement

CSH has filed a patent application on brain stimulation and received honoraria as editor from Elsevier Publishers, Amsterdam. The remaining author declares that the research was conducted in the absence of any commercial or financial relationships that could be construed as a potential conflict of interest.
