# Supplementary material for: Absence of Alpha-tACS Aftereffects in Darkness Reveals Importance of Taking Derivations of Stimulation Frequency and Individual Alpha Variability Into Account
Source: Front Psychol. 2018 Jun 20;9:984. doi: 10.3389/fpsyg.2018.00984 (PMC6019480; doi:10.3389/fpsyg.2018.00984)
Supplement: Supplementary file 1 [file Image_1.pdf]

Supplementary Material

Absence of alpha-tACS aftereffects in darkness reveals importance of taking derivations of stimulation frequency and individual alpha variability into account

Heiko I. Stecher[1], Christoph S. Herrmann[1,2*]

[1] Experimental Psychology Lab, Department of Psychology, European Medical School, Cluster for excellence "Hearing for all", Carl von Ossietzky University, Oldenburg, Germany

[2] Research Center Neurosensory Science, Carl von Ossietzky University, Oldenburg, Germany

*** Correspondence:**
Prof. Dr. Christoph S. Herrmann

Experimental Psychology Lab,

Carl-von-Ossietzky-Universität Oldenburg,

Ammerländer Heerstraße 114-118,

2611 Oldenburg, Germany

Tel.: +49 441 798 4936

Fax: +49 441 798 3865

Email: christoph.herrmann@uni-oldenburg.de

1 Supplementary Figures

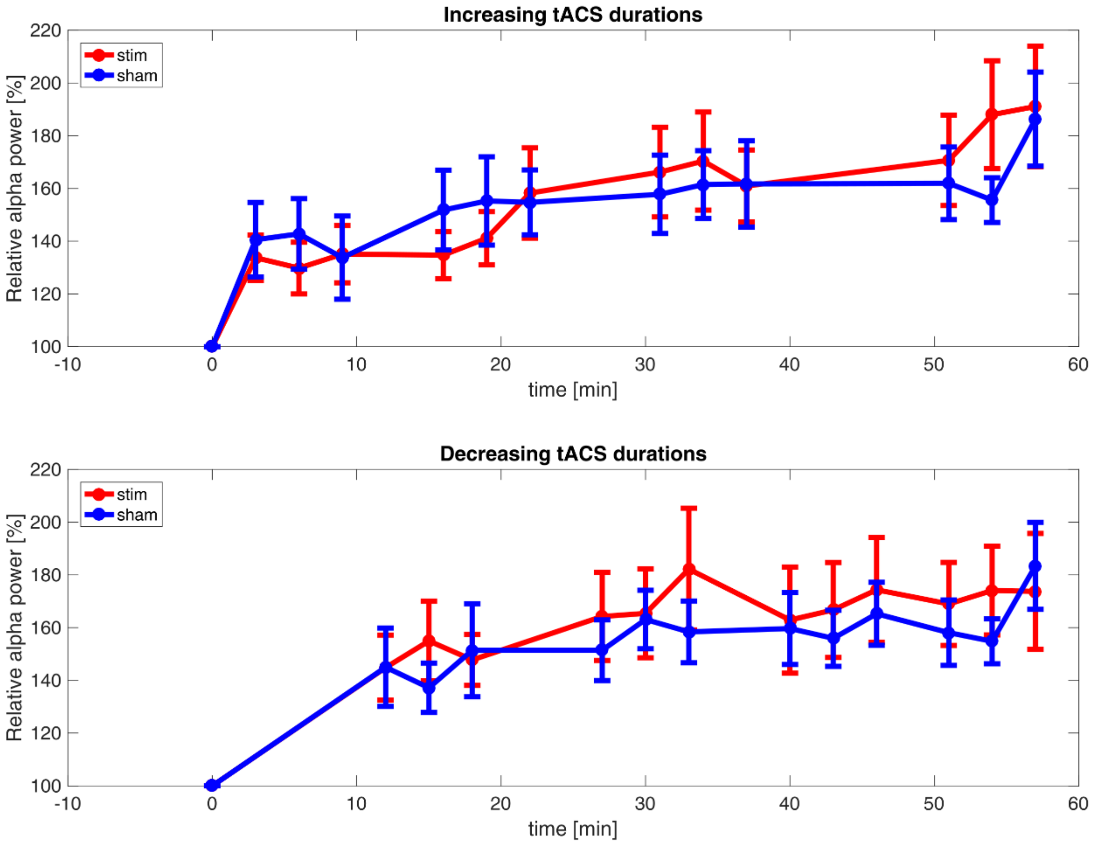

Supplementary Figure 1**.** Relative parietal α-power post stimulation

(A) Time-course of α-power relative to baseline, comparing increasing-sequence stimulation group (red) and sham (blue). Each point represents the average power of a 3-min observation window. Error bars depict the standard error of the mean. (B) Time-course of α-power relative to baseline, comparing decreasing-sequence stimulation group (red) and sham (blue): Each point represents the average power of a 3-min observation window. Error bars depict standard error of the mean.

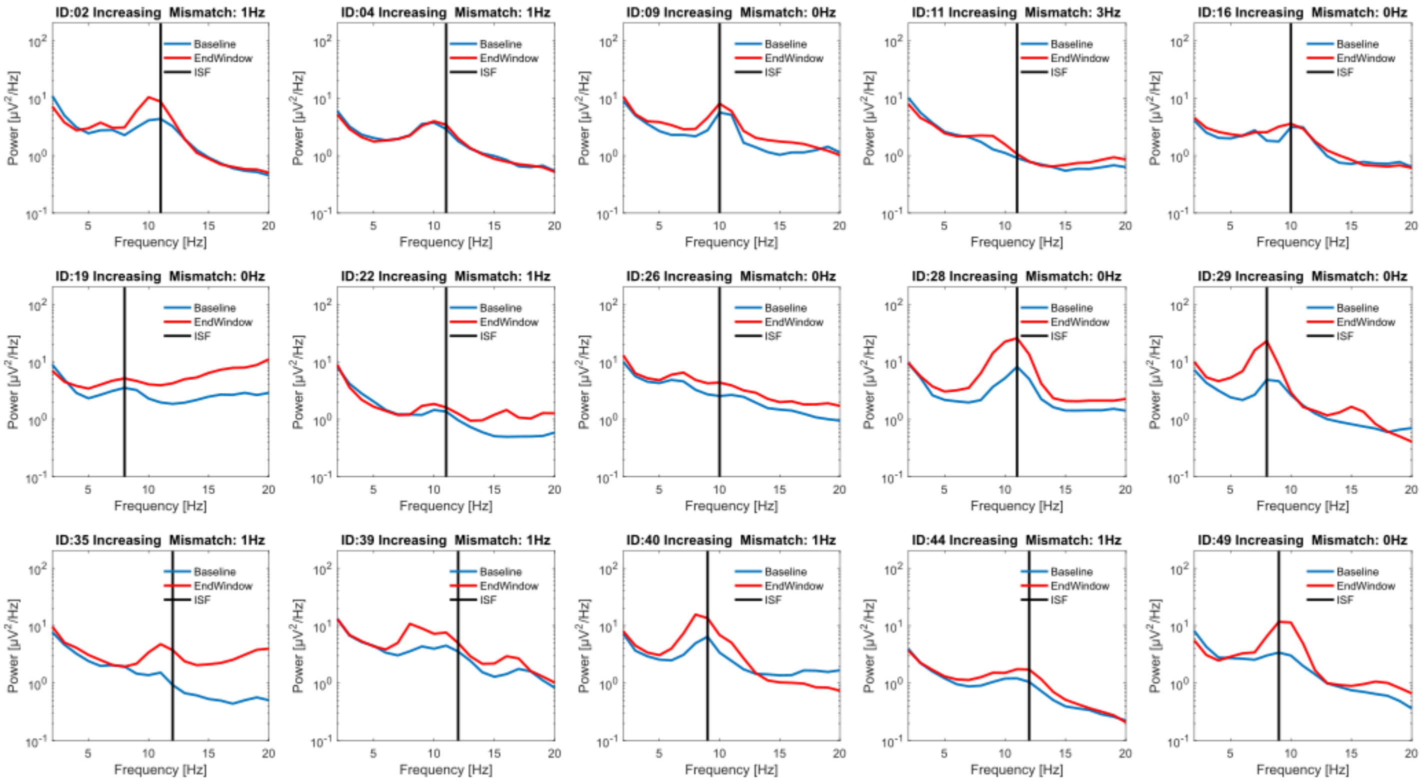

Supplementary Figure 2. Posterior power spectra of all participants of the increasing-stimulation group are shown. The blue lines depict the power in the 3 min baseline before the first stimulation block, the red line depicts the power spectra in the last 9 min of the experiment. The black line marks the Individual stimulation frequency, that was determined before the start of the experiment from a raw 3 min recording of Pz. The mismatch between the IAF peak of the last 9 mine recording and the ISF is given in each subtitle.

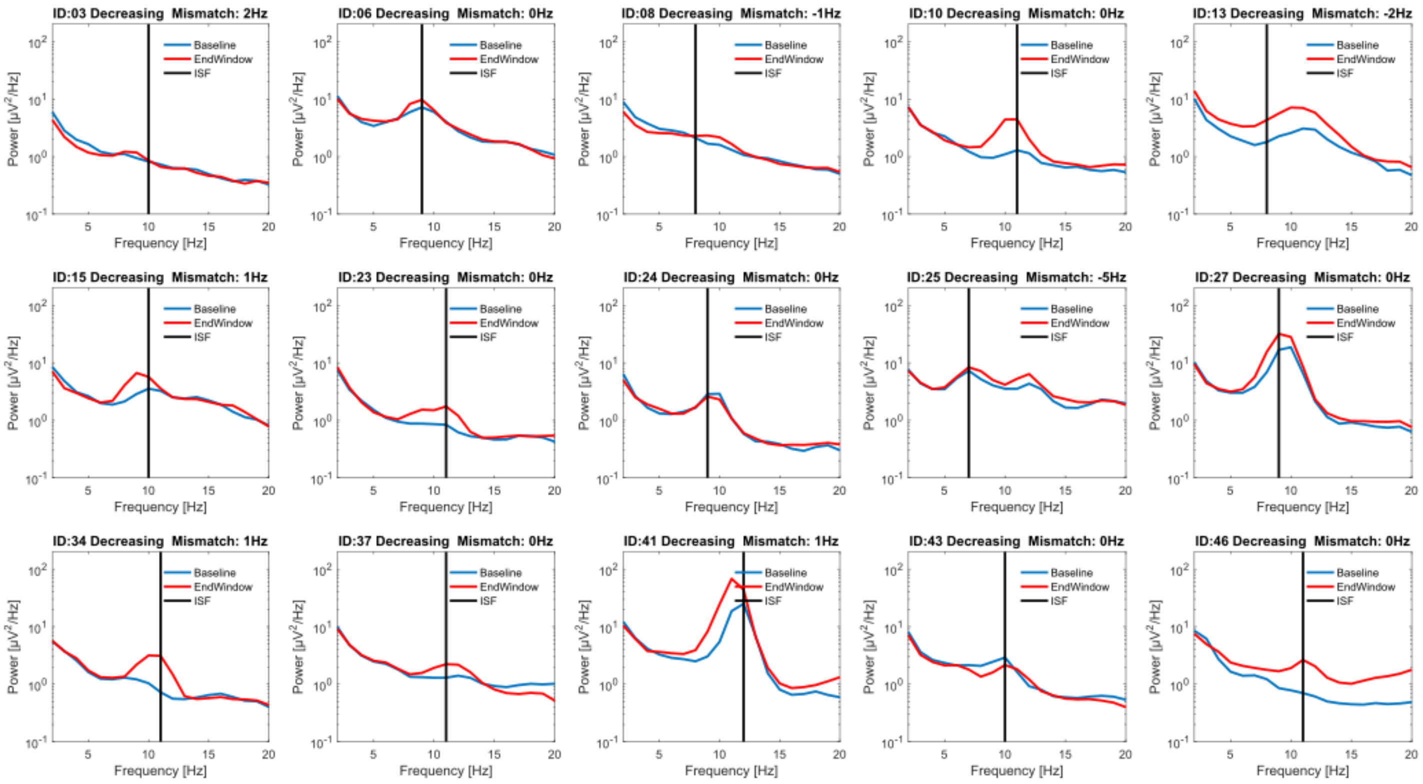

Supplementary Figure 3. Posterior power spectra of all participants of the decreasing-stimulation group are shown. The blue lines depict the power in the 3 min baseline before the first stimulation block, the red line depicts the power spectra in the last 9 min of the experiment. The black line marks the Individual stimulation frequency, that was determined before the start of the experiment from a raw 3 min recording of Pz. The mismatch between the IAF peak of the last 9 mine recording and the ISF is given in each subtitle.

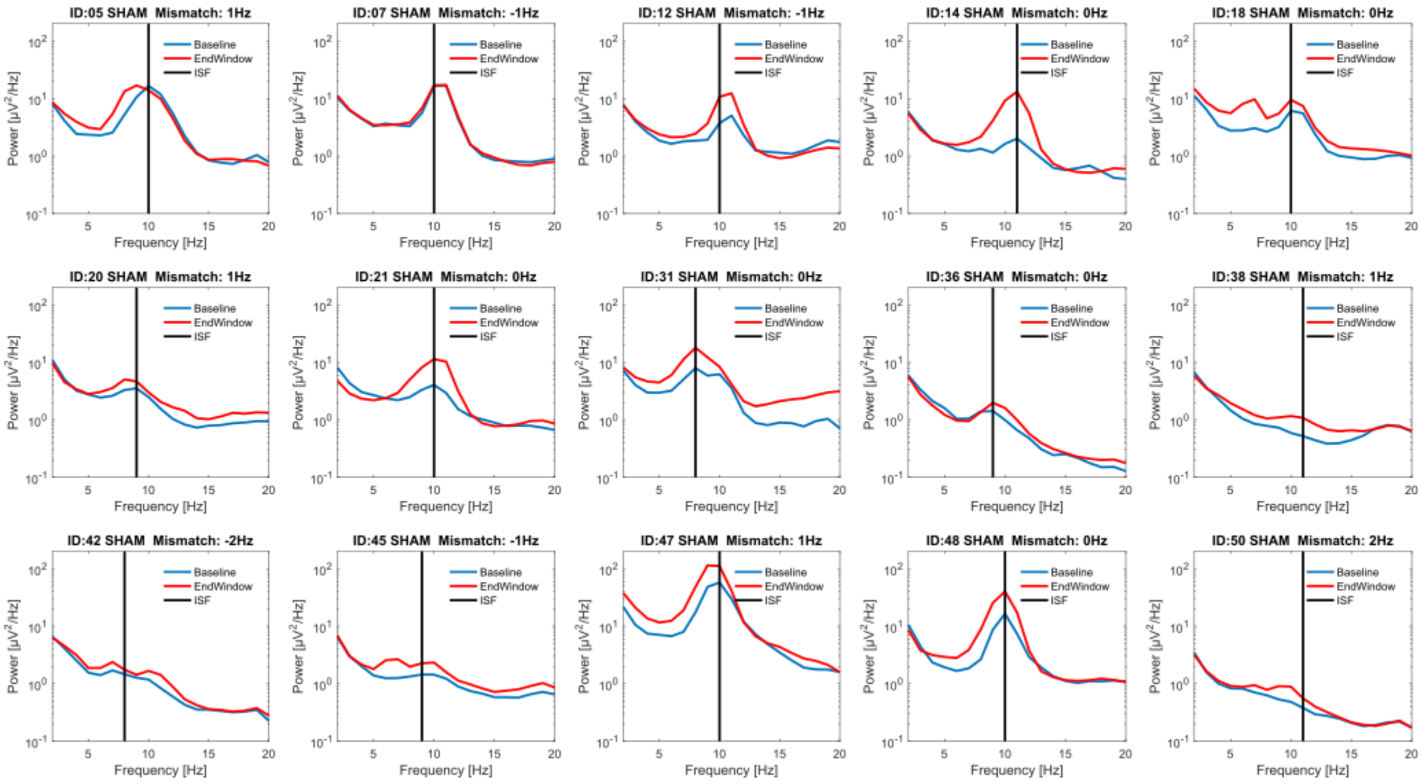

Supplementary Figure 4. Posterior power spectra of all participants of the sham-stimulation group are shown. The blue lines depict the power in the 3 min baseline before the first stimulation block, the red line depicts the power spectra in the last 9 min of the experiment. The black line marks the Individual stimulation frequency, that was determined before the start of the experiment from a raw 3 min recording of Pz. The mismatch between the IAF peak of the last 9 mine recording and the ISF is given in each subtitle.
